# Investigation of radiomics based intra-patient inter-tumor heterogeneity and the impact of tumor subsampling strategies

**DOI:** 10.1038/s41598-022-20931-z

**Published:** 2022-10-14

**Authors:** T. Henry, R. Sun, M. Lerousseau, T. Estienne, C. Robert, B. Besse, C. Robert, N. Paragios, E. Deutsch

**Affiliations:** 1 Molecular Radiotherapy and Therapeutic Innovation, Paris-Saclay University, Gustave Roussy, Inserm 1030, 94800 Villejuif, France; 2 Department of Medical Imaging, Gustave Roussy, 94805 Villejuif, France; 3 Department of Radiation Oncology, Gustave Roussy, 114 Rue Edouard Vaillant, 94805 Villejuif, France; 4 Mathematics and Informatics for Complexity and Systems (MICS), Paris-Saclay University, CentraleSupélec, 91190 Gif-sur-Yvette, France; 5 Department of Medical Oncology, Gustave Roussy, 94805 Villejuif, France; 6 Molecular Predictors and New Targets in Oncology, Paris-Saclay University, Gustave Roussy, Inserm 981, 94800 Villejuif, France; 7 TheraPanacea, Paris, France

**Keywords:** Cancer imaging, Biomarkers

## Abstract

While radiomics analysis has been applied for localized cancer disease, its application to the metastatic setting involves a non-exhaustive lesion subsampling strategy which may sidestep the intrapatient tumoral heterogeneity, hindering the reproducibility and the therapeutic response performance. Our aim was to evaluate if radiomics features can capture intertumoral intrapatient heterogeneity, and the impact of tumor subsampling on the computed heterogeneity. To this end, We delineated and extracted radiomics features of all visible tumors from single acquisition pre-treatment computed tomography of patients with metastatic lung cancer (cohort L) and confirmed our results on a larger cohort of patients with metastatic melanoma (cohort M). To quantify the captured heterogeneity, the absolute coefficient of variation (CV) of each radiomics index was calculated at the patient-level and a sensitivity analysis was performed using only a subset of all extracted features robust to the segmentation step. The extent of information loss by six commonly used tumor sampling strategies was then assessed. A total of 602 lesions were segmented from 43 patients (median age 57, 4.9% female). All robust radiomics indexes exhibited at least 20% of variation with significant heterogeneity both in heavily and oligo metastasized patients, and also at the organ level. None of the segmentation subsampling strategies were able to recover the true tumoral heterogeneity obtained by exhaustive tumor sampling. Image-based inter-tumor intra-patient heterogeneity can be successfully grasped by radiomics analyses. Failing to take into account this kind of heterogeneity will lead to inconsistent predictive algorithms. Guidelines to standardize the tumor sampling step and/or AI-driven tools to alleviate the segmentation effort are required.

## Introduction

In the race for accessible, non-invasive and generic biomarkers, radiomics^1,2^, the extraction and analysis of a high number of features from imaging data, has emerged as a promising approach. From routinely obtained medical images, the radiologist or radiation oncologist delineates the patient's tumor and its digital signature is extracted. Using the digital footprint of tumors, radiomics analysis has the potential to unravel what is hidden from the trained eyes of an experienced radiologist: the objective quantification of the image-based heterogeneity of the whole tumor and its significance^3,4^. In contrast, standard biopsies are invasive, time and money consuming, and can only access a small random fraction of the complete tumor, making tissue-based heterogeneity assessment incomplete at best, misleading at worst^5^.

**Figure 1:**
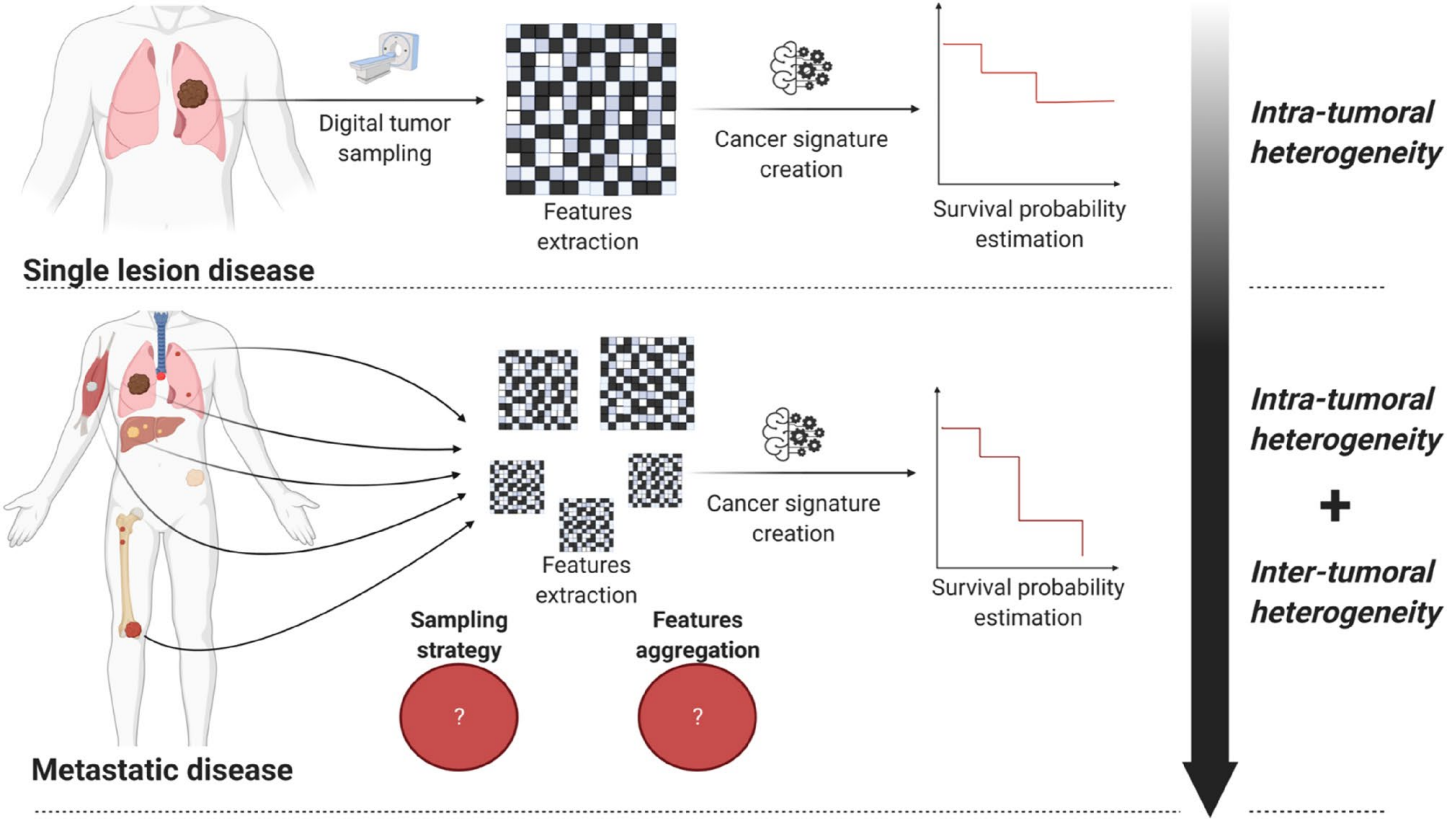
Radiomic analysis strategy: from one lesion radiomics to metastatic disease data extraction. While the analysis pipeline for single lesion disease is now described, the metastatic disease pipeline still has some grey areas: first there is no existent guidelines for the sampling strategies of tumors (which one? how many?), and second, there is no consensus on how to aggregate the extracted features for further analysis. Indeed, the number of lesions delineated per patient could vary, and the downstream machine learning pipeline used for single lesion analysis cannot be reused «as-is». Created with BioRender.com.

Some promises of radiomics, such as completeness and cost-efficiency, can be fulfilled in its initial scope of application: local or loco-regional cancer, with a limited number of lesions, ideally only the primitive tumor. But, extending the scope to metastatic disease, where the total number of lesions per patient can be greater than one hundred, can be challenging. While spatial heterogeneity at the tumor level is of great interest in the localized cancer setting^6^, heterogeneity between patient’s lesions is also a quantity to measure in the metastatic setting^7,8^. Indeed, polyclonality is a key factor of treatment resistance^9,10^, making any biomarker of clonal heterogeneity a game-changer for patient’s care. Immune checkpoints inhibitors (ICI) have further emphasized such consideration given the high rate of non-binary response pattern at the patient level^11^. Thus, the following question arises: can radiomics analysis be performed for metastatic disease in the same way as for localized disease? In light of the critical difference between heterogeneity at a specific tumor site, and between different tumor deposits, the logical answer would be to exhaustively segment and analyse all tumors of each patient. Yet, top cited radiomics research in the metastatic setting from 2010 to 2020 has chosen another path (Supplementary Table), mimicking what is usually done in RECIST evaluation^12^, or sampling only one lesion per patient, potentially missing an entire level of heterogeneity (Fig. 1).

The aim of our study was to quantify image-based heterogeneity captured by radiomics features as a function of the number of tumor lesions analyzed, and to evaluate the degree of information loss induced by commonly used subsampling strategies.

## Results

### Experimental design overview

**Figure 2:**
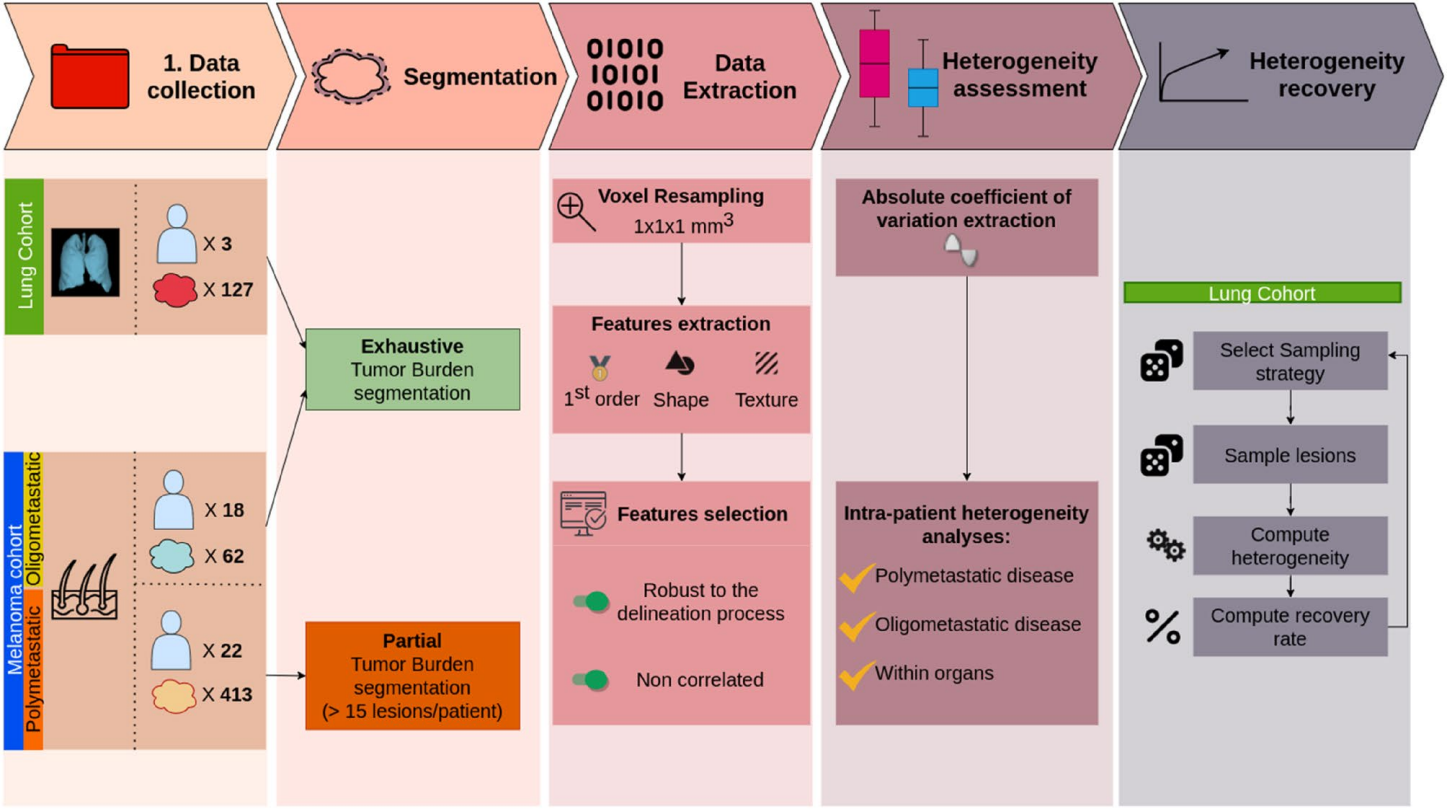
General view of the data pipeline and the analysis process.

As a first step, we explored intrapatient heterogeneity captured by radiomics in the discovery cohort of patients with metastatic lung cancer (cohort L), both at the patient’s level and at the patient’s organ level. A sensitivity analysis was then performed using only uncorrelated radiomics features robust to the delineation process. To confirm the insights gained from cohort L, the same analysis was validated on a larger validation cohort of patients diagnosed with metastatic melanoma patients (cohort M), with a particular focus on oligometastatic patients (below 5 visible tumors in total) and highly metastatic patients (more than 15 visible tumors in total). Finally, to better grasp the amount of heterogeneity lost by commonly used tumor subsampling strategies during the delineation process, we crafted two global heterogeneity measurements, and performed a heterogeneity recovery analysis. The experimental design of the study is illustrated in Fig. 2.

### Participants

Of the four patients manually explored with available portal venous contrast enhanced computed tomography of thorax, abdomen and pelvis (CT TAP IV+) in cohort L, three were included in the study (patient A 63 years-old male, B 58 years-old female, and C 38 years old male), and one excluded given his low number of lesions. A total of 127 lesions were segmented, each patient exhibiting more than 25 lesions. The most frequent lesion location was the lung (total: 92–72.4%). The patient with the highest number of lesions had 69 lesions, with three different metastatic sites, while the maximum number of metastatic sites was five.

Regarding cohort M, 40 patients were included, with a median age of 57 years and 42.5% of females. 22 patients with at least 15 tumor lesions and 18 patients with between 2 and 5 tumor lesions were respectively included in cohorts M-multi and M-oligo, for a total of 475 lesions segmented. The most frequent metastatic location was the lymph node (total 188–39.6%). A complete description of the study population and the lesion distribution across cohorts, patients and metastatic locations is provided in Table 1 and supplementary Figure.

**Table 1:** Description of the study population and of the lesion distribution across cohorts, patients and metastatic locations.

	Overall	Cohort A			Cohort B	
		Patient A	Patient B	Patient C	Oligometastatic (18)	Multimetastatic (22)
n_patients		3			40	
Disease		Metastatic lung cancer			Metastatic melanoma	
Age (median ± IQR)	57 (49–64)	63	58	38	57 (50–64)	
					63 (54–70)	52 (44–59)
sex (F) %	41.9	M	F	M	42.5	
					50.0	57.1
n_lesions	602	127			475	
		30	28	69	62	413
Nature (%)						
Bone	27 (4.5)	18 (60.0)	0 (0.0)	0 (0.0)	9 (2.2)	0 (0.0)
Lung	199 (33.1)	1 (3.3)	24 (85.7)	67 (97.1)	96 (23.2)	11 (17.7)
Lymphangitis	1 (0.2)	0 (0.0)	0 (0.0)	1 (1.4)	0 (0.0)	0 (0.0)
Lymphnode	187 (31.1)	0 (0.0)	4 (14.3)	0 (0.0)	150 (36.3)	33 (53.2)
Peritoneum	31 (5.1)	5 (16.7)	0 (0.0)	0 (0.0)	26 (6.3)	0 (0.0)
Pleural	5 (0.8)	4 (13.3)	0 (0.0)	1 (1.4)	0 (0.0)	0 (0.0)
Soft tissue	41 (6.8)	2 (6.7)	0 (0.0)	0 (0.0)	32 (7.7)	7 (11.3)
Adrenal gland	8 (1.3)	0 (0.0)	0 (0.0)	0 (0.0)	5 (1.2)	3 (4.8)
Liver	96 (15.9)	0 (0.0)	0 (0.0)	0 (0.0)	91 (22.0)	5 (8.1)
Other	7 (1.2)	0 (0.0)	0 (0.0)	0 (0.0)	4 (1.0)	3 (4.8)

### Variation of the radiomics features at the patient and organ level in cohort L

Radiomics features exhibited high variation across lesions for the same patient. Patientwise, the absolute coefficient of variation ranged from 1.6% for the Inverse Difference Moment Normalized (IDMN) to 321% for Skewness, considering all radiomics features. Pooling all features at the patient level, the mean coefficient of variation per patient was respectively 144, 117 and 158% for patient A, B and C.

**Figure 3:**
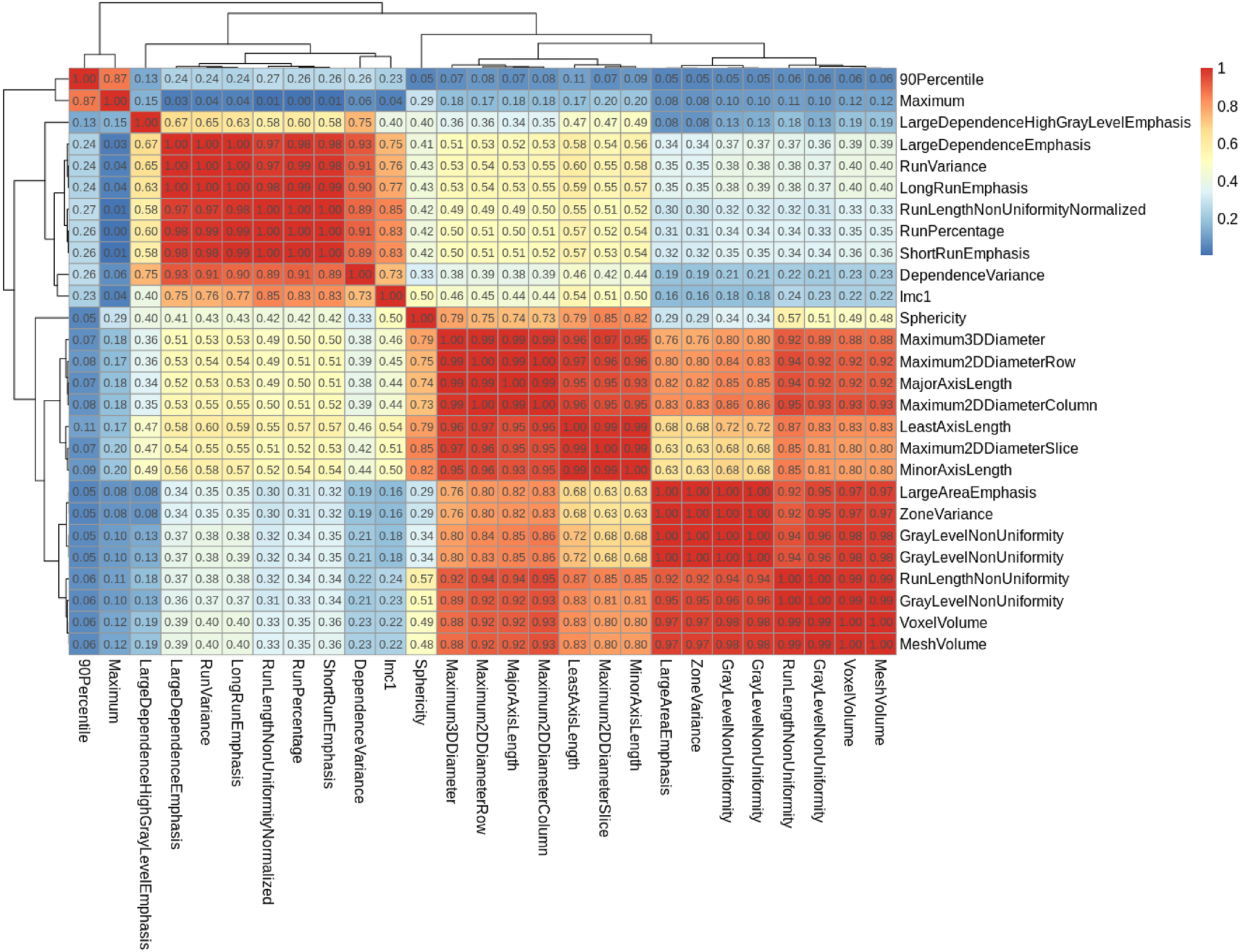
Clustered correlation heatmap of the 27 robust radiomic features. The absolute value of the correlation between features was displayed, for ease of interpretation. 8 clusters were distinguishable, and one radiomic features per cluster were selected for the sensitivity analysis.

After feature segmentation robustness assessment using the concordance correlation coefficient of radiomics indexes between the original and eroded/dilated segmentations, only 27 features exhibited a coefficient above 0.8 and were kept for the correlation analysis. Based on a visual assessment of the clustered correlation heatmap (Fig. 3), eight clusters were identified, and the following robust and uncorrelated features were selected: 90 Percentile, Large Dependence High Gray Level Emphasis, Large Dependence Emphasis (based on lexicographic sorting), Dependence Variance, Informational Measure of Correlation 1, Sphericity, Maximum 2D Diameter Slice (equivalent to RECIST measurement) and Voxel Volume (rather than Mesh Volume due to better computational efficiency). The absolute coefficient of variation ranged from 12% for Sphericity to 687% for Voxel Volume. Pooling this subset of features at the patient level, the mean coefficient of variation per patient was similar to the mean coefficient of variation for all features, respectively equal to 124, 108 and 170% for patient A, B and C.

**Figure 4:**
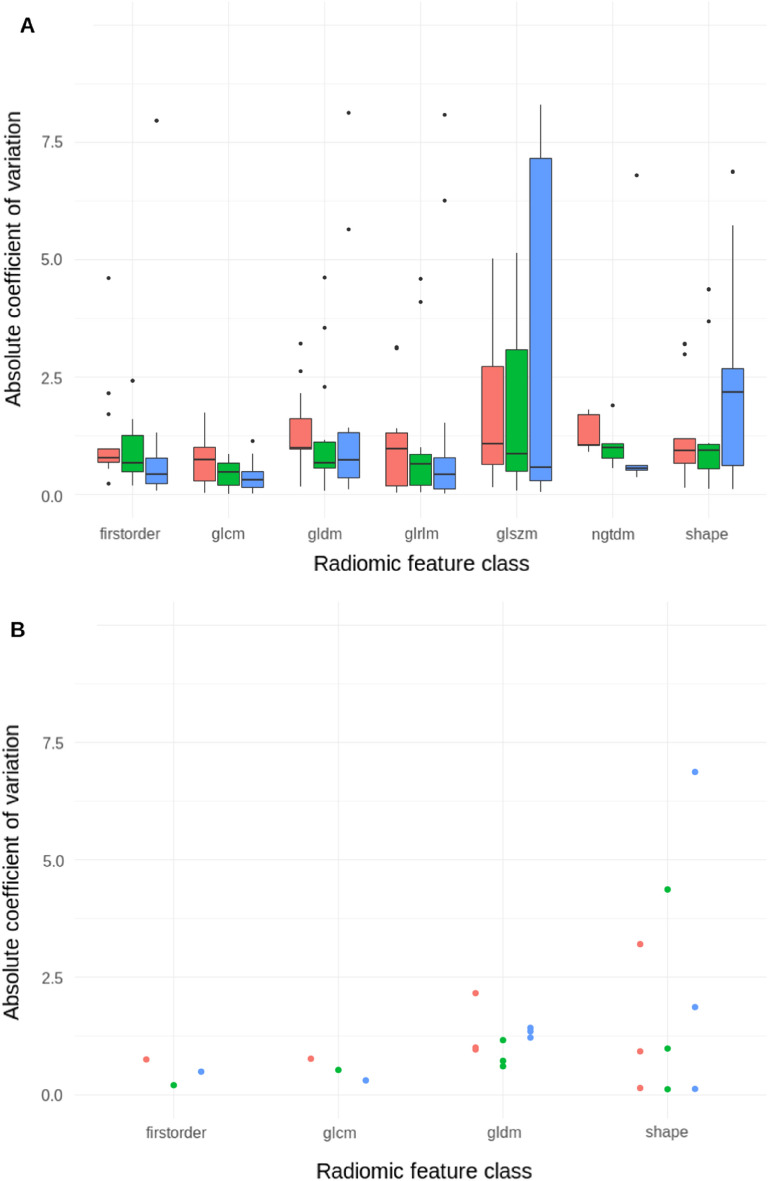
Absolute coefficient of variation considering (**A**) all 107 radiomic features; (**B**) eight robust and uncorrelated radiomic features (Sensitivity analysis) for cohort L. Radiomics features are subdivided by patient (one color per patient) and the commonly used radiomic features’ classes. All radiomics features exhibited high level of variation for each of the three patients, with shape and glszm features being the most affected. Theses levels of variation were similar with the robust and uncorrelated radiomic features’ set. *glcm: Gray Level Co-occurrence Matrix ; gldm: Gray Level Dependence Matrix; glrlm: Gray Level Run Length Matrix; glszm: Gray Level Size Zone; ngtdm: Neighbouring Gray Tone Difference Matrix.*

The distributions of the absolute coefficient of variation across the seven radiomics classes are depicted in Fig. 4 for both all radiomics features, and the robust and uncorrelated subset. Robust and uncorrelated features still exhibited a significant level of variation. Sphericity and Volume variation showed respectively the minimal and maximal variations for all three patients. All robust radiomic indexes, except for Sphericity, exhibited at least 20% of variation.

Finally, going down to the organ level, lung lesions exhibited heterogeneous features, ranging from 21% for 90 percentile to 419% for tumor Volume, for the same patient.

### Analysis of the variation of radiomics features at the patient and organ level in the validation cohort M

To confirm the insights gained from cohort L analysis, We conducted the same analysis on cohort M, using only robust radiomics features. The mean absolute coefficient of variation ranged from 7.2% for Sphericity, to a maximum of 144% for Volume. All features except Sphericity exhibited at least 30% of variation. All radiomics features exhibited variation significantly different from 0 (*p* < *1e−11*). These results were consistent across both oligo and multi metastatic sub-cohort, with mean absolute coefficient variation significantly different from 0% for all radiomics features (*p* < *1e−6* and < *1e−5* in the multi and oligometastatic cohort respectively). Moreover, there was no significant difference in the mean absolute coefficient of variation between oligo and multi metastatic population for seven out of eight of the robust radiomics features (Voxel Volume, Informational Measure of Correlation 1, Large Dependence Emphasis, Dependence Variance, Sphericity, Maximum 2D Diameter Slice, 90 Percentile).

Finally, focusing on patients with more than one lesion for either lymph node, liver, or lung, radiomic features were highly unstable, ranging from 6% for sphericity to 106% for Volume. Advanced textural features like Large Dependence High Gray Level Emphasis or Dependence Variance exhibited an absolute variation of 54 and 18% respectively for intra-patient intra-liver lesions.

### Lesion similarity exploration and sampling simulation study

**Figure 5:**
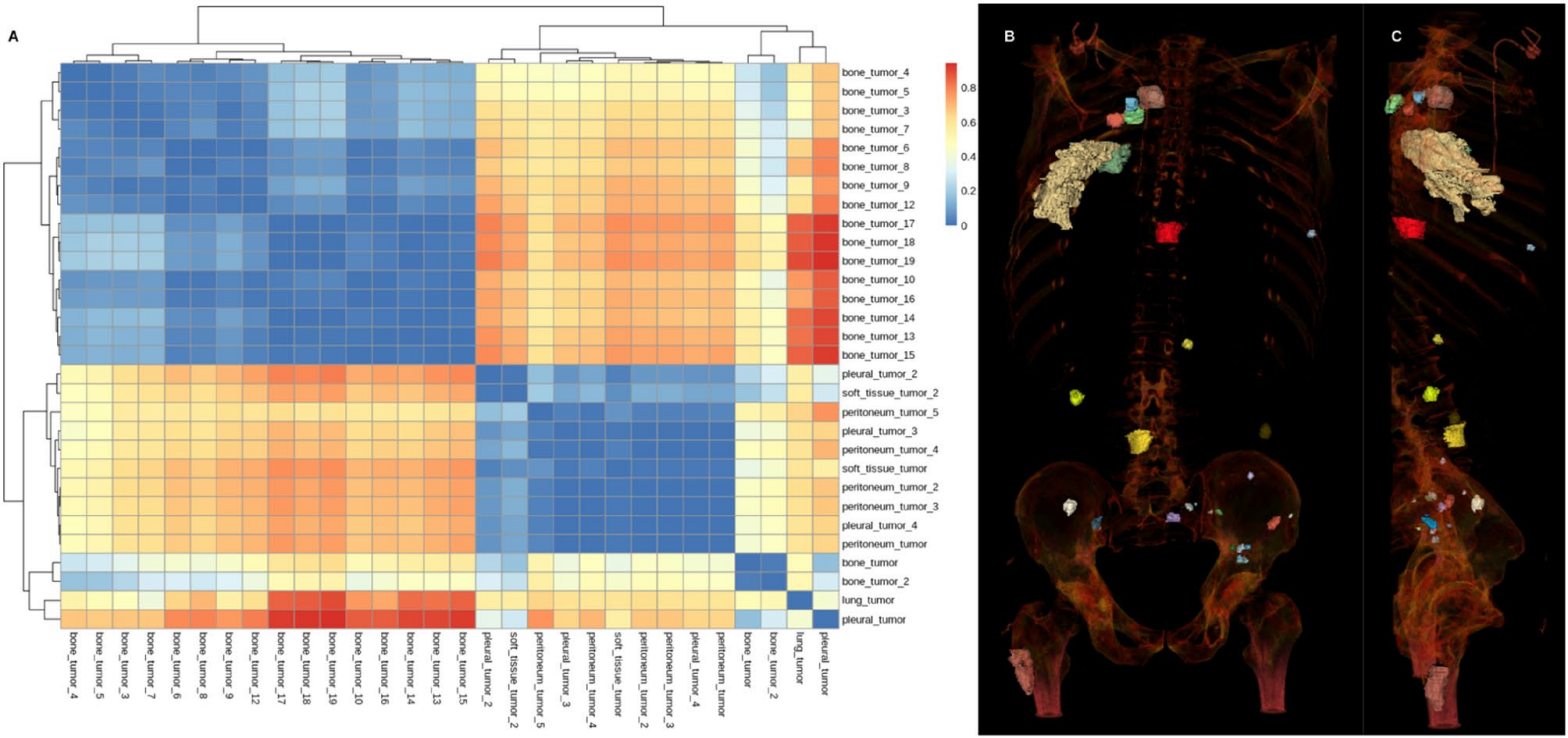
(**A**) Dissimilarity between lesions’ pairs for patient A. (**B**) and (**C**) Volume rendering of the contoured lesions (face + profile). Patient A suffered from bone, lung, pleural peritoneal and soft tissue tumors. Most bone tumors clustered together but showed high dissimilarities with non-bone tumors and even with a small cluster of two bone lesions. There was no obvious clustering of the non-bone lesions (pleural, lung and peritoneal lesions), even when the lesions arised from the same tissue (e.g. pleural lesions).

We computed the pairwise dissimilarity between all pairs of lesions for each patient in cohort L. To visualize the dissimilarity between lesions for each patient, we clustered the patient lesions solely based on the dissimilarity matrix previously computed (cf Appendix). An example of distance distribution is visualized in Fig. 5. Then, we summarized this distribution using the two previously defined heterogeneity indexes. The maximal tumoral divergence was respectively 0.94, 0.92 and 0.91 (maximal value: 1.0), and the average tumoral heterogeneity was 0.39, 0.21 and 0.21 (maximal value: 1.0).

Using these heterogeneity indexes, we explored the captured heterogeneity of common subsampling techniques. The case where only one lesion per patient was sampled was trivial: it is impossible to compute pairwise dissimilarity for only one lesion, and this sampling strategy completely failed to capture inter-tumoral intra-patient heterogeneity. For all other sub-sampling strategies, heterogeneity indexes were clearly below the ground truth value obtained by exhaustive tumor sampling. Ground truth values, mean estimated values and their 95% CI obtained by bootstrapping can be found in Table 2.

**Table 2:** Percentage of heterogeneity recovered by each tumor sampling strategy (mean values and their 95% confidence interval obtained using 500 simulations for each).

	Exhaustive ground truth value	2 lesions at random	3 lesions at random	2 lesions per site at random	3 lesions per site at random	2 lesions per site at random, excluding bone lesions (RECIST like)
Average tumoral heterogeneity	0.27	48% [7–93]	67% [30–100]	19% [7–33]	22%[11–33]	19% [4–33]
Maximal tumor divergence	0.92	27% [5–55]	55% [22–67]	11% [3–18]	16% [9–24]	10% [3–18]

Using completely random sampling strategies yielded better estimates of the true heterogeneity indexes than sampling by metastatic site or RECIST-like sampling strategies: sampling three lesions at random recovered 67% (95% CI: [30–100]) and 55% (95% CI: [22–67]) of the average tumoral heterogeneity and maximal tumor divergence respectively, whereas sampling three lesions per metastatic site recovered 22% (95% CI: [11–33]) and 16% (95% CI: [9–24]) respectively. When sampling completely at random, increasing the number of sampled lesions improved the heterogeneity index estimate, while empirically this was not the case for sampling by metastatic location. Yet, none of the sub sampling methods were able to recover the true maximal tumor divergence.

**Figure 6:**
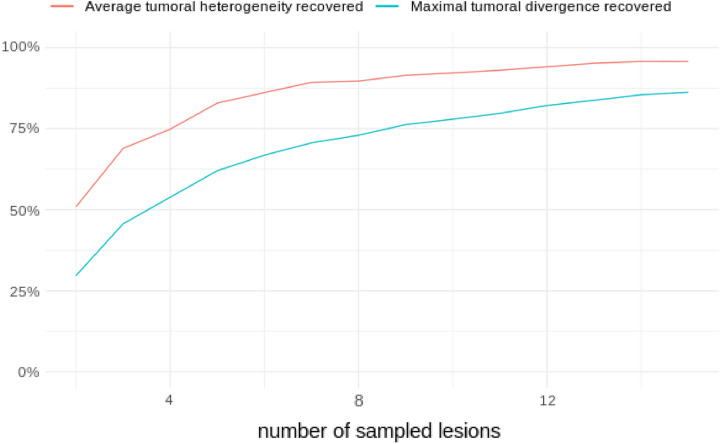
Recovery curves of maximal tumoral divergence and average tumoral heterogeneity for cohort L. Commonly used subsampling strategies select up to 3–4 lesions, failing to correctly capture intra-patient inter-tumor heterogeneity. Even when the delineation effort is scaled up to 15 lesions, none of the two heterogeneity indexes are fully recovered.

Based on the empirical values of maximal tumoral divergence and average tumoral heterogeneity, more than eight lesions per patient would have been necessary to recover at least 75% of the true heterogeneity captured by the whole lesion distribution analysis (Fig. 6).

## Discussion

In the era of personalized medicine, radiomics studies have drawn attention, promising to predict treatment response and survival duration, leveraging the hidden “big data” from imaging studies, surpassing trained imaging specialists. But machine learning comes with its own set of strengths and potential pitfalls^13^. Through a series of in-silico experiments, we put in perspective what is achievable and what has been foreseen in previous works. First, using a cohort of 602 contoured lesions across 43 different patients and two different cancer types, we demonstrated that radiomics features vary significantly between tumors from the same patient. To prevent a strong correlation between radiomics indexes to lead us to a spurious conclusion, we performed the same analysis on a very small segmentation robust and uncorrelated subset of radiomics features, and found that all of them except one exhibited at least 30% of variation. This level of heterogeneity was corroborated by cohort M, where it was observed not only in heavily metastasized patients, but also in oligo metastasized ones. Finally, tumor heterogeneity was also detected at the organ level: tumors from the same patient, and the same host organ still exhibited heterogeneity that needed to be accounted for. This has both positive and negative implications. On the bright side, radiomics features can indeed capture inter-tumoral heterogeneity, making them promising candidates for imaging based heterogeneity biomarkers. Finding image based heterogeneity between lesions of the same patient is most likely to have a significant impact on patient’s care: in the era of ICI, where pseudoprogression, hyper progression or dissociated progression are all a reality^14^, being able to link a tumor-centric signature to a tumor response pattern would be an invaluable insight for patient treatment, e.g. by adding local treatment for the predicted non responding tumors. This is a significant paradigm shift, the key unit of interest for response would not only be the patient, but also each of its individual tumor deposits, allowing for an even finer granularity in the treatment response assessment, and treatment options decision.

On the other hand, such levels of variation question the sturdiness of previous radiomics studies carried out on metastatic patients. Indeed, if not all tumor deposits have the same digital signature for the same patient, any predictive machine learning algorithm based on radiomics features would give a different output depending on the tumor considered as input. Thus, the only way to be able to trust such a predictive algorithm would be to define a priori completely deterministic and reproducible rules for lesion sampling.

To build on these findings, we crafted two simple heterogeneity indexes that combine robust and uncorrelated radiomics features from an arbitrary number of lesions into a patient-level descriptor. While there is no guarantee that these indexes correlate with common clinical endpoints like progression-free or overall survival, they have the advantage of being constructed without modeling prior, in an unsupervised fashion. They are also good metrics to evaluate how much heterogeneity is lost when applying a priori rules for lesion sampling. Our simulations demonstrated that metastatic disease cannot be treated the same way as localized disease. Common subsampling techniques either completely missed inter-tumor intra-patient heterogeneity (one lesion sampling), or greatly underestimated its extent. In cohort L constituted of completely annotated patients, the empirical value of lesions to annotate for recovering at least 75% of the true heterogeneity would have been 8. This is in stark contrast with what has been done in previously published studies (Supplementary Table).

Our study comes with some limitations. First, we do not provide any conceptual or empirical proof that image-based and histological based heterogeneity are well correlated, nor that image based heterogeneity is predictive of therapy response, progression-free survival, or overall survival. There are already multiple works linking radiomics features to histology and/or immunochemistry features^3,15,16^, While obviously the ultimate goal, we argued that it was necessary to take the time to first carefully consider the implications of the metastatic setting, before heading straight for biological correlation and predictive performance. As such, we found that not all radiomics indexes might be relevant for heterogeneity estimation. For example, sphericity was the least variable robust radiomics index in both cohorts L and M, which is in line with common radiological knowledge: a rounded nodule of variable size is a common radiological sign of metastasis^17^. This feature is most likely to be irrelevant for heterogeneity assessment. Another limitation is the small number of patients included, despite a high number of lesions annotated. Nonetheless, our main goal was first to prove the existence of tumor heterogeneity at the imaging level, and second, roughly estimate its importance. As for the first goal, only one patient would have been sufficient. As for the second goal, we found that all of the 43 patients exhibited significant radiomics index variation. All patient imaging studies were made before immunotherapy treatment, and patients were pre-treated with at least one line of chemotherapy before imaging, which could have increased the observed tumoral heterogeneity. As such, our findings might not be relevant to untreated freshly diagnosed metastatic patients. Moreover, lung cancer and melanoma are known for high tumoral heterogeneity, and radiomics studies for other types of cancer might not be as sensitive to the sampling process^18^. Further work on other cancer types might be needed to confirm our findings. Meanwhile, as a precautionary measure, conclusions drawn from highly heterogeneous cancer types should be transposed to others. Finally, demonstrating that exhaustive tumor sampling is required for accurate heterogeneity estimation is rather cumbersome, as exhaustive tumor segmentation comes with unaffordable trained-radiologist time consumption. As such, two orthogonal future directions can be taken to build upon this work: either searching for better tumor subsampling technique, based on visual analysis of the radiological images; or looking for more efficient ways to perform complete tumor detection and segmentation. AI-driven tools might be a good fit for this job^19,20^.

In conclusion, image-based inter-lesions heterogeneity at the patient level can be successfully grasped by radiomics analyses. Further guidelines and AI-driven tools to standardize and alleviate the tumor sampling and segmentation task are required. Future works should account for the variability between tumors in the same patient, and link it to relevant biological and clinical endpoints.

## Methods

### Study design and data collection

Two different cohorts (cohort L and M) were collected. Patients with metastatic lung cancer referred to our institution for ICI treatment between 2012 and 2019 were considered for inclusion in cohort L. For each patient, radiological examinations between 3 months before and 1 week after the first cure were automatically retrieved from the picture archiving and communication system (PACS) using in-house software. Clinical data for those patients were manually collected from the electronic health records (EHR) generated during patient follow-up. Then, using lexicographic order, consecutive patient’s imaging studies were manually explored, and only patients with a single acquisition pre-treatment CT TAP IV+ were investigated. Patients with less than ten visible lesions were excluded from the cohort. All visible lesions of each patient were contoured for this cohort.

To confirm the findings discovered in cohort L, cohort M was collected from patients with metastatic melanoma referred to our institution treated with anti-PD-1 or anti-PD-L1 (monotherapy or combination) from May 1st, 2012 to February 18, 2019 with similar requirements for imaging and clinical data. Given the annotation cost, not all tumors in a patient were annotated, except for oligometastatic patients (cohort M-Oligo). Lesions in cohort M were annotated by residents, checked and corrected if necessary by an experienced radiation oncologist (R.S). Only patients with oligometastatic disease from two to five lesions (cohort M-oligo), and multi-metastatic patients with at least 15 annotated lesions (cohort M-multi) were included. Cohort M-multi was used to validate the extent of radiomics feature variation at the patient scale, while cohort M-oligo was used to assess if inter-tumoral heterogeneity at the patient’s scale was also a reality for oligometastatic disease. Finally, in light of the previous result, we also checked if intra-organ intra-patient tumor heterogeneity was captured by radiomics features.

### Lesion delineation and radiomics features extraction

Lesion detection can be an easy task, with detection speed roughly proportional to the experience of the medical reader. However, lesion segmentation, the act of extensively delineating the tumor contours, does not scale well with practice, and requires a tremendous time and focus from the practitioner.

Given the high annotation cost of exhaustive tumor delineation and the exploratory nature of our work, we decided to use a minimum number of lesions rather than a minimum number of patients to manage the segmentation effort. The minimum number of lesions to collect was set to 100 overall for this cohort. Accordingly, all visible tumors for each patient in cohort L were delineated, regardless of their size or location, using the 3D Slicer software^21^ (https://www.slicer.org/). More specifically, lesions that would be deemed non targetable as per RECIST 1.1 were segmented, including bone lesions, and lesions with a greatest diameter below 1.5 cm. The segmentation process was performed by the same senior nuclear radiologist (T.H.) for all patients, in order to remove variability from inter-observer delineation.

Tumor segmentations for patients from cohort M were performed by radiation oncology residents, using Raystation Software version 9B (RaySearch Laboratories, Stockholm, Sweden). Each annotation was validated by a senior radiation oncologist (R.S) and a senior nuclear radiologist (T.H.).

For both cohorts, to ensure that the observed variability was only due to tumor heterogeneity at the patient level, each patient of each cohort was delineated by only one annotator using only one software.

Imaging data and tumor segmentations were resampled to a common 1 × 1 × 1 mm^3^ voxel size using B-spline interpolation, and the following 107 radiomics features were extracted using the pyradiomics^22^ package version 3.0: 18 first order (FO) statistics, 14 shape-based (SB) features, 24 Gy level co-occurrence matrix (GLCM) features, 16 Gy level run length matrix (GLRLM) features, 16 Gy level size zone matrix (GLSZM) features, five neighbouring gray tone difference matrix (NGTDM) features, and 14 Gy level dependence matrix (GLDM) features. Before radiomics extraction, image discretization was performed using a fixed bin width of ten, as done in previous studies^3^.

### Data analysis

To quantify the raw captured heterogeneity, the absolute coefficient of variation (CV) of each radiomics index was calculated at the patient level. A sensitivity analysis was then performed using only a subset of all extracted features as advised previously^23^. First, only features robust with respect to the segmentation process were kept: each tumor segmentation was eroded and dilated isotropically by 1 mm and radiomics indexes that exhibited a concordance correlation coefficient below 0.8 between the original segmentation and each of the derived ones were dropped. Then, we performed a correlation analysis: radiomics features were clustered using hierarchical clustering, and only one feature per identified cluster was selected, keeping only the most understandable one (e.g., tumor volume rather than gray level non-uniformity index), or the first one by lexicographic sorting if the correlated features were all hard to conceptualize.

To confirm the insights gained from the extensively annotated cohort L, the same analysis was performed on cohort M-oligo and M-multi, and then at the patient’s organ level for lung, liver and lymph nodes, using only the robust radiomics features. The mean CV coefficient of each radiomics feature was tested for non-nullity using a one sample t-test on the M cohort. Mean CV coefficients of each radiomics feature were compared between cohorts M-oligo and M-multi using the Wilcoxon rank sum exact test. A Bonferroni correction for multiple testing was performed and accordingly a p-value below 0.0015 was deemed significant. All statistical analyses were performed using the R software.

Finally, as a first step towards formal heterogeneity estimation at the patient level, using the extensively annotated cohort L, we proposed two overall heterogeneity measurements with the previously selected features: the maximal tumoral divergence, defined as the maximum pairwise dissimilarity between the patient’s tumor deposits, and the average tumoral heterogeneity, defined as the average pairwise dissimilarity (detailed formulation in Appendix). To account for the high dimensionality of the radiomics feature vector of each tumor, we chose the cosine distance as the dissimilarity metric. These heterogeneity measurements were used to assess the extent of information lost by commonly used tumor sampling strategies. The sampling strategies explored were: two or three lesions randomly selected; random sampling of a maximum of two or three lesions per metastatic location; sampling two lesions at most per metastatic location excluding bone lesions (RECIST 1.1 like sampling strategy). For each sub-sampling strategy, 500 simulations were conducted, and the mean value of each heterogeneity index and its 95% confidence interval (IC 95) was computed. Finally, we performed random lesion sampling of two to a maximum of fifteen lesions per patient, in order to empirically determine the number of lesions required to recover at least 75% of the average tumoral heterogeneity and maximal tumoral divergence.

The complete data pipeline and analysis process is depicted in Fig. 2.

### Ethical approval

The study was performed in accordance with relevant guidelines and regulations. Waiver of informed consent was granted for the specific details of this study according to French law regulation (MR-004) and the General Data Protection Regulation (GDPR), given the retrospective and non-interventional nature of this study. The study protocol was approved by the Gustave Roussy medical ethics committee (IRB n° 2021-85).

## Supplementary Information

Supplementary Information.

## Data Availability

In accordance with the General Data Protection Regulation (GDPR), none of the individual participant imaging data can be made available to others. However, the deidentified radiomic features dataset that underlie the results reported in the article will be made available to researchers who provide a methodologically sound proposal beginning 3 months and ending 2 years following article publication. Information regarding submitting proposals and accessing data may be submitted to eric.deutsch@gustaveroussy.fr.
