# Supplementary material for: Investigation of radiomics based intra-patient inter-tumor heterogeneity and the impact of tumor subsampling strategies
Source: Sci Rep. 2022 Oct 14;12:17244. doi: 10.1038/s41598-022-20931-z (PMC9568579; doi:10.1038/s41598-022-20931-z)
Supplement: Supplementary file 1 — Supplementary Information. [file 41598_2022_20931_MOESM1_ESM.docx]

Supplmentary materials

**Suppementary Table.** *Sampling strategy and rate of the top 5 most cited journal articles as ranked by Scopus between 2010 and 2020 using the following query: TITLE-ABS ( radiomics AND metastatic AND response ) AND PUBYEAR > 2009 AND PUBYEAR < 2021 AND ( LIMIT-TO ( DOCTYPE , "ar" ) ) AND ( LIMIT-TO ( SUBJAREA , "MEDI" ) ).*

| **Author** | **Title** | **Sampling strategy** | **Sampling rate (per patient)** |
| --- | --- | --- | --- |
| [Trebeschi, S. et al (2019)](https://doi.org/10.1016/j.tranon.2016.01.008) | Predicting response to cancer immunotherapy using noninvasive radiomic biomarkers | Measurable lesions defined as any tumor lesion (primary or metastatic) whose entire border could be identified. Lesions with Ill-defined borders or poorly visualized were excluded | 3 (median) |
| [Antunes, J. et al (2016)](https://doi.org/10.1016/j.tranon.2016.01.008) | Radiomics analysis on FLT-PET/MRI for characterization of early treatment response in renal cell carcinoma: A proof-of-concept study | Primary tumor lesion | 1 |
| [Dohan, A. et al (2020)](https://doi.org/10.1136/gutjnl-2018-316407) | Early evaluation using a radiomic signature of unresectable hepatic metastases to predict outcome in patients with colorectal cancer treated with FOLFIRI and bevacizumab | Contouring of the dominant liver lesion (DLL) | 1 |
| [van Helden, E. J. et al (2018)](https://doi.org/10.1007/s00259-018-4100-6) | Radiomics analysis of pre-treatment [18F]FDG PET/CT for patients with metastatic colorectal cancer undergoing palliative systemic treatment | All visually identifiable tumour lesions defined as SUVpeak higher than two times the SUVmean of the blood pool, with a minimum volume of 4.2 mL | 4 (mean) |
| [Dercle, L. et al (2020)](https://doi.org/10.1093/jnci/djaa017) | Radiomics Response Signature for Identification of Metastatic Colorectal Cancer Sensitive to Therapies Targeting EGFR Pathway | Target liver lesions selected per RECIST 1.1 criteria | 4 (median) |

**Supplementary Figure: Flow chart of the patient selection process**


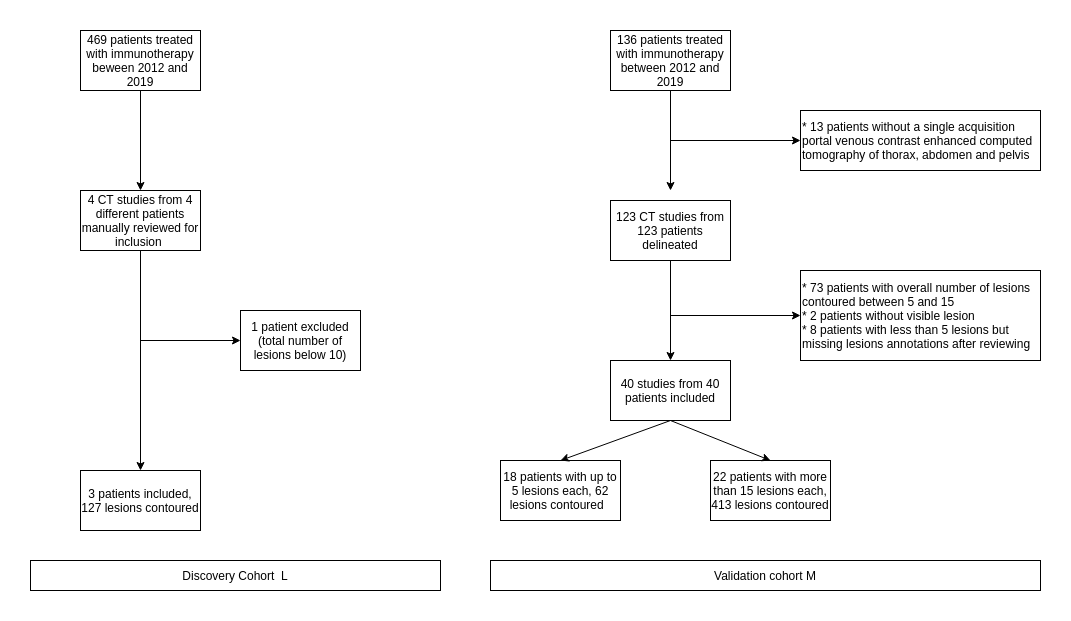


**Detailed formulation of the average tumoral heterogeneity and the maximal tumor divergence.**

Given N the total number of lesions of a patient, and J the number of radiomic features involved in the computation, we define the matrix X ∈ ℝ^NxJ^ as the digital signature of a patient’s tumor burden.

Given that radiomic features have very different ranges of value, we first normalize the matrix feature wise, dividing each column of the matrix by the absolute value of its mean, effectively making each column having zero-mean, but preserving its respective variance.

Then we compute the cosine dissimilarity between each possible pair of lesions. As an example, given a pair of lesions (A,B), it is defined as:

$cosinesimilarity\left( A,B \right)=\frac{A\cdot B}{\left| \left| A \right| \right|x\left| \left| B \right| \right|}$

$cosinedissimilarity\left( A,B \right)=1-cosinesimilarity\left( A,B \right)$

Finally, given all cosine dissimilarity values for each possible pair of lesions, we define the average tumoral heterogeneity as the average of all cosine dissimilarity values, and the maximal tumor divergence as the maximal cosine dissimilarity value.
